# Why Chromosome Palindromes?

**DOI:** 10.1155/2012/207958

**Published:** 2012-07-15

**Authors:** Esther Betrán, Jeffery P. Demuth, Anna Williford

**Affiliations:** Department of Biology, University of Texas at Arlington, Box 19498, Arlington, TX 76019, USA

## Abstract

We look at sex-limited chromosome (Y or W) evolution with particular emphasis on the importance of palindromes. Y chromosome palindromes consist of inverted duplicates that allow for local recombination in an otherwise nonrecombining chromosome. Since palindromes enable intrachromosomal gene conversion that can help eliminate deleterious mutations, they are often highlighted as mechanisms to protect against Y degeneration. However, the adaptive significance of recombination resides in its ability to decouple the evolutionary fates of linked mutations, leading to *both* a decrease in degeneration rate *and* an increase in adaptation rate. Our paper emphasizes the latter, that palindromes may exist to accelerate adaptation by increasing the potential targets and fixation rates of incoming beneficial mutations. This hypothesis helps reconcile two enigmatic features of the “palindromes as protectors” view: (1) genes that are not located in palindromes have been retained under purifying selection for tens of millions of years, and (2) under models that only consider deleterious mutations, gene conversion benefits duplicate gene maintenance but not initial fixation. We conclude by looking at ways to test the hypothesis that palindromes enhance the rate of adaptive evolution of Y-linked genes and whether this effect can be extended to palindromes on other chromosomes.

## 1. Evolution of Sex-Limited Chromosomes 

### 1.1. Evolution of Sex-Limited Chromosomes-Theory

**Figure 1:**
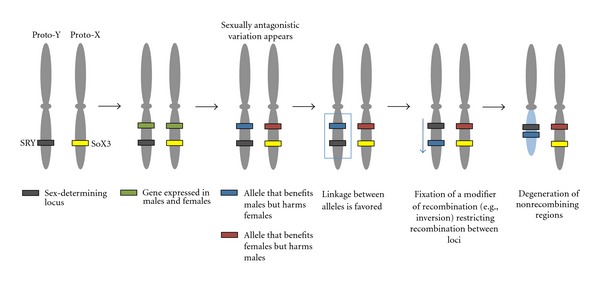
The model of sex chromosome evolution. Close linkage between sexually antagonistic variation and the sex-determining gene has been proposed to start Y chromosome morphological differentiation from the X chromosome.

Sex-limited chromosomes are unique in that they often have a small, peculiar gene content [1–5]. Classically, sex chromosomes are thought to originate from a pair of autosomes in three phases: (1) one homolog acquires a sex determining factor; (2) selection favors linkage between sexually antagonistic variants and the sex determination factor, thereby reducing or eliminating regional recombination; (3) the forces of mutation, drift, and selection in regions of low recombination lead to rapid gene loss (Figure 1; [6–8]). To the extent that this model is true, *positive selection *for reduced recombination (e.g., selection to fix chromosomal inversions and/or other modifiers of recombination [9, 10]) is responsible for providing the spark that ignites proto-Y chromosome morphological differentiation from the proto-X chromosome (Figure 1).

**Figure 2:**
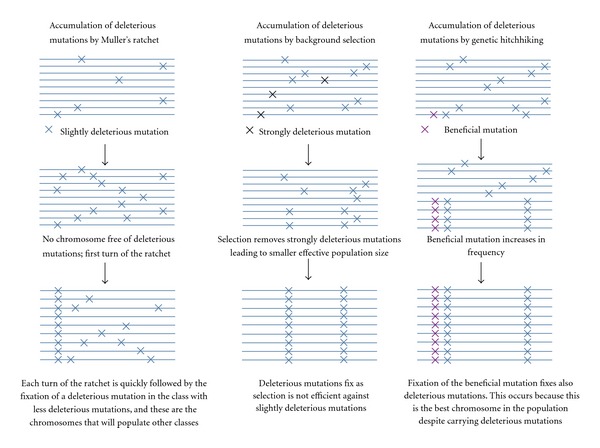
(a) The three processes that lead to degeneration of the Y chromosome: Muller's ratchet (see [109] for details of how every turn of the ratchet is followed by fixation of a deleterious allele), background selection, and genetic hitchhiking. Only in the case of genetic hitchhiking, the fitness of the Y chromosome increases through time.

In the third phase of sex chromosome differentiation, three different processes—Muller's ratchet, background selection, and genetic hitchhiking—may contribute to degeneration of the Y (or W) chromosome once recombination is reduced in all or part of the nascent sex-specific chromosome [11–13]. These three mechanisms are instances of the general Hill-Robertson effect that describes the reduction in the efficiency of selection in the presence of segregating mutations under selection when recombination is either absent or reduced [14–16]. Muller's ratchet will operate when deleterious mutations occur, and the class of Y chromosomes with the least deleterious mutations is lost from the population by drift and cannot be recovered because of the lack of recombination. Background selection will lead to the fixation of weakly deleterious mutations due to the reduction in effective population size brought about by the selection against strongly deleterious mutations in regions with reduced recombination. Genetic hitchhiking will occur when a beneficial mutation drags along the fixation of deleterious mutations in the nonrecombining region of the Y chromosome. The long-term consequences for Y chromosome fitness are very different for each of these processes (Figure 2). The first two processes make the fitness of Y chromosomes worse on average as time goes by while genetic hitchhiking improves the Y on average. Interestingly, these processes have different likelihood of operating at different times in the process of Y chromosome differentiation. Muller's ratchet and background selection are predicted to be strong degenerating forces when there are many genes on the nonrecombining region of the Y, while genetic hitchhiking will dominate the nonrecombining region of the Y when the genic content is smaller [17, 18]. Thus, genetic hitchhiking is predicted to be the dominant process on older Y chromosomes that have small gene content. In addition, since the cessation of recombination often occurs in strata [10, 19, 20] and consequently a limited number of genes are involved in each bout of degeneration, Bachtrog [17] proposed that genetic hitchhiking might actually play an important role throughout the chromosome's degeneration.

The relative contribution of the above mechanisms to the evolution of the Y chromosome is difficult to assess as neither the rate of mutations under selection nor the distribution of their fitness effects are well known. In addition, the fitness effects of Y-linked mutations might differ depending on how and when Y inactivation and dosage-compensation evolve. X- and Y-linked loci are expected to differ in fitness either because more beneficial mutations can fix on the X than on the Y [21] or because more deleterious mutations fix on the Y than on the X [13]. This will generate selection pressure for transcriptional downregulation of the Y-linked loci and upregulation of the X-linked loci. If such Y inactivation and dosage compensation occur, subsequent mutations in dosage compensated regions may not be deleterious anymore but rather neutral or sometimes beneficial (i.e., if they facilitate the silencing of the maladapted Y-linked genes). The fraction of Y-linked genes that are now neutral or whose inactivation can be beneficial could be large if dosage compensation occurs “block by block” [22]. Recent studies of systems with young sex chromosomes suggest that gene silencing might be an early step in Y chromosome degeneration [23] and dosage compensation may evolve concomitantly with Y chromosome degeneration [24]. That is, much of the Y degeneration might be a neutral or even adaptive process [18, 21, 22].

### 1.2. Evolution of Sex-Limited Chromosomes—Data

 In humans, most of the approximately 1,000 genes present on the X chromosome are absent from the Y chromosome [25]. Apart from the 29 genes that are present within the recombining regions of the X and Y chromosomes (i.e., the pseudoautosomal regions), the male specific region of the Y chromosome (MSY) contains only 19 genes that can be traced to ancestral autosomes (Table 1; [25, 26]). Similar extensive gene loss from the Y is seen in other mammalian lineages, where the number of extant genes that originated on the protosex chromosomes does not exceed ~20 genes [27, 28]. Independently evolved sex chromosomes in other taxa, including birds, snakes, plants, and insects, followed similar patterns of gene loss after recombination ceased on the sex-limited chromosome (Y or W; [19, 20, 29–31]).

Interestingly, the pattern of gene loss in humans and other lineages (including species where females are the heterogametic sex) suggests that phases 2 and 3 of Y (or W) chromosome evolution recur multiple times, generating a series of strata with different levels of degeneration relative to the X (or Z) [10, 19, 20, 31]. The pattern of rapid gene loss following stratum formation and subsequent stabilization of gene content [26] is consistent with temporal dynamics of the evolutionary forces implicated in the degeneration of the Y chromosome [30, 32]. Furthermore, among the major lineages of birds, the same pair of ancestral autosomes independently proceeded through phases 2 and 3 [33]. These two patterns support the idea that, once the process of sex chromosome differentiation initiates, the presence of strong sexually antagonistic variation will drive the chromosomes through similar steps and to convergent ends in independent lineages. 

Recent sequencing of primate Y chromosomes has uncovered what might be called the 4th phase of sex chromosome evolution characterized by gene preservation and Y chromosome specialization through acquisition and amplification of genes with testis expression [26, 34, 35]. Inter- and intraspecific sequence comparisons suggest that purifying selection on the Y chromosome is strong enough to prevent the full decay of genes that originated on the protosex chromosomes. Analyses of gene loss in three primates—human, chimpanzee, and rhesus macaque—indicates that lineage-specific gene losses in the human and rhesus MSY are restricted to the stratum that most recently ceased to recombine with the X, while the few genes in older strata (1–4) have been conserved by purifying selection for more than 25 million years [26, 36]. While no lineage-specific gene losses were detected in gorilla, the chimpanzee MSY has lost 5 ancestral genes since splitting from the human lineage ~6 million years ago being the only lineage that shows instability among primates thus far (Table 1; [26, 36, 37]). Conservation of gene content is also found outside primates. At least 6 Y-linked X-degenerate genes specific to marsupial lineage have been preserved for ~50 million years [38]. Polymorphism data also supports the efficient retention of some genes by purifying selection. Within human populations, analysis of sequence variation in 16 Y-linked single-copy X-degenerate genes indicates efficient purifying selection, finding little difference in the protein sequence among males [39].

In addition to the preservation of the X-degenerate genes, Y chromosomes show clear signs of differentiation through lineage-specific gene gain. In humans, 80% of genes on the MSY (60 out of 78) are members of 9 gene families (Table 1). Some of these families originated by duplication of X-degenerate genes (TSPY, RBMY, and HSFY), but other families arose through gene duplication and subsequent amplification of autosomal genes (DAZ and CDY) while others possibly originated de novo on the Y (PRY and BPY2) as no X-linked or autosomal homologues have been identified [26, 34, 40–43]. Two single-copy genes (TGIF2LY and PCDH11Y) were also recently acquired by the human Y via translocation of 3.4 Mb from the X chromosome [34]. New genes are added to the Y chromosome in other mammals as well. For example, studies of MSY in horse identified 17 novel and acquired genes that are also present on the donkey Y but are absent in other mammalian Y chromosomes [28]. New gene families have been independently gained on the bovine and carnivore MSY through translocation of autosomal gene blocks followed by amplification [44–46]. In *Drosophila  melanogaster*, gene acquisition plays a major role in the evolution of the Y chromosome as all protein-coding genes (<20) result from duplication of autosomal genes [47, 48]. These findings favor the view that Y chromosome gene content is not merely characterized by degeneration. Rather, it is much more dynamic than previously recognized, having evolutionary stages that vary dramatically in gene birth and death rates.

It is notable that the vast majority of the genes that have been amplified or acquired on the Y chromosome in different lineages are expressed predominantly or exclusively in testis and have spermatogenesis-related functions [26, 28, 34–36, 44, 46, 49–55]. In mammals, the testis-specific expression of amplified and acquired genes contrasts the much broader expression profile of single-copy X-degenerate genes (see Table 1 for primate examples). Such acquisition and retention of different testis-specific genes in different lineages suggest that specialization for male-fertility functions is a driver of Y chromosome evolution.

### 1.3. Models for the 4th Phase of Y Evolution

There are several models invoking positive selection to explain Y chromosome gene content that are consistent with Y-linked genes being a lasting and important determinant of male fitness. First, phase 2 of the classical model introduced above suggests that positive selection favoring tight linkage between sex determining loci and those with sexually antagonistic variation starts the differentiation and degeneration of the Y (Figure 1; [7]). While the classical model explains the emergence of a sex-specific gene (i.e., a sexually antagonistic gene that becomes Y linked) linked to sex determination factors on the Y, sexual antagonism also provides a framework to explain recruitment of new Y-linked genes. For instance, intralocus sexual conflict on autosomes can be resolved by duplicating the allele benefiting males onto the Y chromosome [56, 57]. A likely example of this model includes sexually selected loci in guppies. Selection in male guppies to make them more attractive to females has been proposed to be so strong that it leads to the Y-linkage of traits that are likely costly in females [58] although it is unclear whether these genes moved to the Y chromosome or they evolved *in  situ*. An alternative resolution of intralocus sexual antagonism that more drastically reshuffles sex chromosome gene content is achieved by invasion of a new male determining gene linked to the male benefiting allele as proposed for a cichlid fish [59]. This particular example follows a previously proposed model related to the resolution of sexual antagonism that involves the turnover of sex determination genes [60].

In addition to the role of sexual antagonism, strong epistasis between Y-linked and X- or autosomal genes could also impact Y chromosome gene content [61]. Beneficial Y-X or Y-autosome combinations will experience positive selection for genomic rearrangements that result in tight linkage. In the case of Y-X epistasis, this could favor the spread of nonrecombining regions as observed in Y chromosomes with multiple strata of differentiation. Y-autosome epistasis would favor duplications of the autosomal genes to the nonrecombining portion of the Y where their linkage would no longer be disrupted. Consistent with this model, the Y chromosome of *D.  melanogaster* shows strong epistatic variance for fitness [61]. The Y chromosome that is the best in one genetic background is worst in another contributing nothing to additive genetic variation for fitness in males [61]. Furthermore, introgressions of *Drosophila * Y chromosomes into different conspecific [62] or heterospecific [63] genomic backgrounds result in misexpression of more than 100 X- and autosomal genes.

Additional models that may govern Y chromosome gene content likely include efficient sex-limited selection, selfish genetic elements, and subfunctionalization. The fact that most Y-linked genes have rapidly evolving sex-specific functions (e.g., only expressed in testes) is a clear indication that sex-limited selection on a haploid chromosome is a large determinant of what remains and/or is duplicated to the Y [64]. The evolution of selfish elements could also explain the Y linkage of some genes. One kind of selfish elements is segregation distorters (i.e., selfish systems that increase in frequency because they bias their transmission to the next generation). In *Drosophila*, one RNA gene family on the Y chromosome (*suppressor of stellate*) has been proposed to be a gene that acquired Y-linkage under positive selection as it acts to suppress *Stellate* expression that has been proposed to be a X-Y selfish segregation distorter [65]. Finally, Koerich and colleagues (2008) also considered that neutral duplication of a testis gene followed by chance loss of the parent copy or the neutral duplication of a broadly expressed gene followed by subfunctionalization could explain some of the gene gains observed for the *Drosophila* Y chromosome [47]. 

Several studies of DNA sequence divergence have suggested the action of positive selection in some Y(W) genes. Gerrard and Filatov [66] studied three genes in 12 mammalian species and concluded that two of them (*USP9Y* and *UTY*) evolved under positive selection. The basis for the selection of these genes is not clear as both of them are broadly transcribed among tissues (Table 1; [66, 67]). Another example, *DAZ*, might also have evolved under positive selection in humans and in this case is easier to explain owing to testes specific expression of the gene [68]. Signatures of positive selection have also been found in female specific W genes. For instance, the *HINTW* gene is under positive selection on the W chromosome of birds [69] and has sex-specific functions in the developing female urogenital tract and ovaries. In plants, Marais et al. [70] analyzed seven Y-linked genes in *Silene* *latifolia *and revealed patterns of divergence in two of these genes (*SlssY* and *DD44Y*) that are consistent with positive selection. 

In addition to analyses of substitution patterns across taxa, polymorphism data has also been analyzed for a few Y chromosome systems. A signature consistent with ongoing positive selection was found on the neo-Y chromosome of *D.  miranda* [71]. However, it is likely that adaptation is, at most, restricted to a few loci and that the faster accumulation of amino acid substitutions and unpreferred codons on the neo-Y compared to neo-X chromosome is the result of reduced efficiency of purifying selection on the nonrecombining neo-Y [32, 72]. Particular models of background selection that include interference between strongly negatively selected sites are also compatible with this polymorphism data [73]. So a population analysis of the fitness effects of these chromosomes is needed to distinguish among the models. In *Silene*, polymorphism is reduced on the Y but it is unclear whether background selection or genetic hitchhiking with beneficial mutations (or both) contribute to the observed reduction [74, 75]. Human Y polymorphism data reveals very low levels of polymorphism on the Y and have been taken as evidence of the small effective population size that accompanies nonrecombining Y chromosome degeneration [76]. However, a more detailed look reveals that reduced variation is mainly due to gene conversion in ampliconic regions [76].

In sum, positive selection may not only be the spark that ignites Y (or W) chromosome differentiation in phase 1 but also continues to influence Y (or W) chromosome evolution in phases 2 through 4, leading to degeneration of some genes due to genetic hitchhiking and possibly the addition of others by duplications and translocations. In the following, we propose that palindromes and amplicons that seem to originate on the Y and W chromosomes late in the process of sex chromosome differentiation might be important chromosomal mutations whose primary role could be to increase the rate of incoming beneficial mutations and accelerate adaptation in old sex chromosomes. 

### 1.4. Y(W) Palindromes

The assembly of the Y chromosome of humans, chimpanzee, and rhesus macaque revealed surprising sequence heterogeneity of the Y chromosome with a substantial portion of these chromosomes occupied by large repeat units, referred to as amplicons [26, 34, 35]. Ampliconic sequences can be organized as tandem arrays as well as palindromes (inverted repeats). The amplicons are extensive, comprising 45% (10.2 Mb) of the euchromatic portion of the MSY in humans, 57% (14.7 Mb) in chimpanzee, and 5% (0.5 Mb) in rhesus macaque [26, 34, 35]. Compared to X-degenerate sequences (i.e., orthologous single-copy X-Y sequences), ampliconic sequences have a higher density of genes and pseudogenes but markedly lower density of retrotransposable elements [34, 35]. 

Palindromes are the most impressive feature of the Y chromosome. These structures are made up of inverted repeats (palindrome arms) separated by a nonduplicated spacer. The length of each arm varies among the three primate species, ranging between 73 kb in rhesus and 344 kb in humans on average [26]. Rhesus macaque has 3 palindromes that occupy about 87% (437 kb) of the ampliconic region, while chimpanzee and humans have 19 and 8 palindromes that make up about 50% (7.5 Mb) and 54% (5.5 Mb) of the ampliconic region, respectively. Twelve of 19 palindromes are specific to chimpanzee lineage [35]. Two of 3 palindromes in rhesus macaque are also found in humans [26] revealing that some palindromes have endured for at least 25 millions of years. 

**Figure 3:**
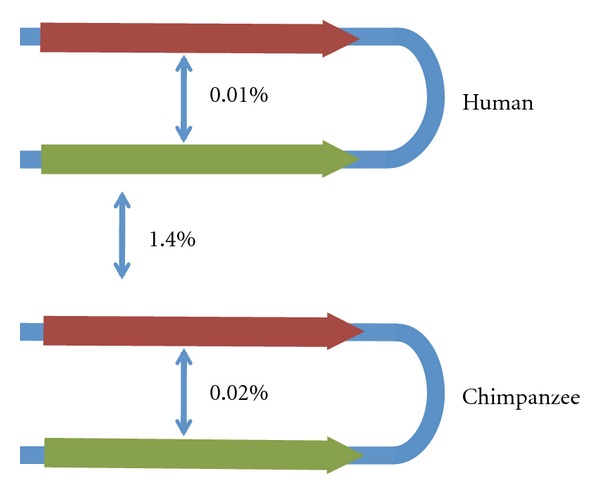
Concerted evolution by gene conversion in primate palindrome 6 [77]: low divergence between paralogs within a lineage but “normal” divergence between orthologs between lineages.

A striking feature of ampliconic MSY regions is the high intrachromosomal sequence identity. In humans, 60% (6.1 Mb) of ampliconic sequences (including all 8 palindromes) show 99.9% or greater intrachromosomal sequence identity. The sequence comparison of the 4 palindromes between humans and chimpanzee revealed that such high sequence identity is maintained by ongoing gene conversion between the arms of the palindromes. Sequence divergence between orthologous palindrome arms was found to be 1.44%, while arm-to-arm divergence within each species is much lower, 0.021% and 0.028% for human and chimpanzee palindromes, respectively (Figure 3; [77]). The rate of gene conversion required to maintain the observed level of sequence identity is estimated to be 2.2 × 10^−4^ per site per generation which means that ~600 duplicated nucleotides have undergone gene conversion between palindrome arms every generation [77]. 

**Figure 4:**
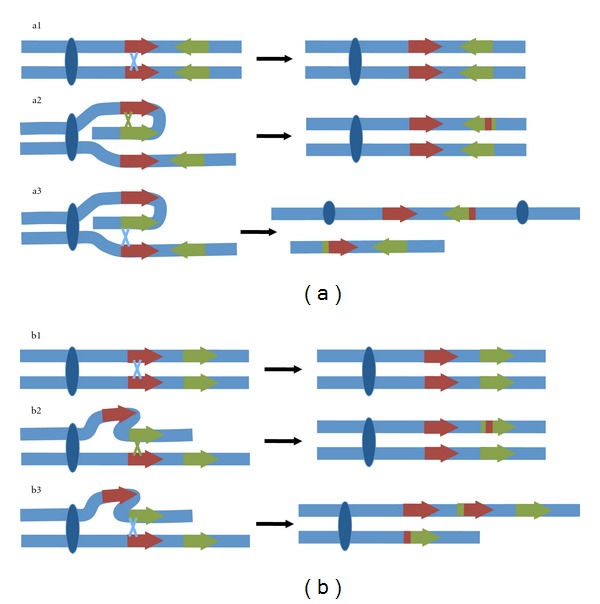
Effects of crossovers (blue lines) and gene conversion (green lines) in Y(W) palindromes (a) and tandem arrays (b). No effect of a crossover is observed if it occurs within the same gene between sister chromatids (a1 and b2). Gene conversion (nonreciprocal transfer of information) is observed if it occurs between different genes within the same palindrome or between tandem duplicates (a2 and b2). Acentric and dicentric chromosomes are produced from a crossover between different genes in palindromes located in sister chromatids (a3). Acentric chromosomes will not segregate properly, and dicentric chromosomes will likely break and lose information when they are pulled to opposite cell poles [78]. Gene gains and losses are produced from a crossover between different duplicates within array located in sister chromatids (b3).

Gene conversion is a standard type of recombination but, unlike crossing over (Figure 4(a) (a1) and Figure 4(b) (b1)), it involves the nonreciprocal transfer of information. This is shown in the central panels of Figure 4 (see Figure 4(a) (a2) and Figure 4(b) (b2); [78]). Gene conversion was first observed as an outcome of allelic recombination (between orthologous sequences of homologous chromosomes) but is now widely recognized as a mechanism of genetic transfer between paralogous sequences ([79] and references therein). The extent of gene conversion is influenced by a number of factors, including sequence identity, physical proximity, and the length of the identical regions [80]. Discovery of ampliconic regions on the primate Y arranged as palindromes and tandem arrays that are expected to promote gene conversion largely changed the view of the Y chromosome from a vestigial part of the genome to a vital chromosome that is capable of escaping the debilitating consequences of the absence of recombination [34, 77]. 

So far, only a few cases of gene conversion on sex-limited chromosomes have been documented outside primates. In the European rabbit, gene conversion occurs between the 23 kb long palindrome arms that house the SRY genes and are 99.94% identical [81]. In galliform birds, multiple tandem copies of W-linked *HINTW* genes undergo gene conversion maintaining high sequence identity between copies within each of the four species studied [82]. A W-linked palindrome in white-throated sparrow shows signs of conversion in a region containing a portion of *CHD1W* intron [83]. Ampliconic regions with large tandem and palindrome-like repeats containing active genes and pseudogenes have been found on the bovine MSY where sequence identity within repeat families ranges from 99.4% to 99.7% [46]. Preliminary analysis of the mouse Y chromosome sequence also identifies multiple palindromes and large repeat units [84]. Whether or not gene conversion is operating in these species awaits further analyses. Despite the current scarcity of information about the detailed organization of most Y chromosomes, data are rapidly accumulating and it is becoming increasingly clear that gene duplication is a common feature of differentiated/old Y chromosomes [28, 46, 51, 84, 85], and we anticipate that more cases of gene conversion will be discovered.

In addition to the possibility of gene conversion, ampliconic structures create an opportunity for ectopic crossing over. For genes located in palindromes, crossing over can occur between gene copies on the same chromatid or between different copies on different sister chromatids. It has been observed that crossover events that involve paralogs from different sister chromatids (Figure 4(a) (a3) and Figure 4(b) (b3)) lead to isodicentric and acentric chromosomes and can result in gene loss and gain [78]. This process may underlie several disease phenotypes in humans including spermatogenesis failure, sex reversal, and Turner syndrome which are associated with inheritance of a rearranged Y and gene loss [78]. Although ectopic recombination does not always lead to reduced male fertility [53, 78, 86, 87], fitness-reducing consequences of ampliconic structure are likely to be frequent enough to impose an upper limit on the number of duplicates that can be maintained in a Y chromosome as a higher number is expected to lead to more ectopic crossovers [88]. Given that palindromes and tandem arrays are fixed in a population and are maintained for long periods of time, the benefits associated with gene duplications must be large enough to offset their deleterious effects.

## 2. Why Chromosome Palindromes? 

The available data suggest that the most important consequence of ampliconic structure relevant to the evolution of Y chromosomes is the opportunity for gene conversion. Some palindromes are very complex in structure and gene content [35, 77, 89] and are maintained for long periods of time (i.e., >25 My in some instances). Palindromic regions also seem to be under purifying selection as it has been observed that transposable elements and retroviruses are removed from the palindromic regions [26, 34]. High levels of intrachromosomal sequence identity are consistent with high rates of ongoing gene conversion within Y(W) chromosome palindromes [26, 35, 77, 81, 82]. In fact, the rate of gene conversion in the palindromes of the human Y chromosome is three orders of magnitude higher than that of human paralogs that are similarly arranged but located elsewhere in the genome [90]. This observation supports the view that the evolution of high levels of gene conversion on the Y chromosome has been favored by selection [76]. Thus, the reason for palindrome emergence and maintenance should be sought in understanding the benefits of gene conversion for the evolution of gene families on the Y chromosome.

The consequences of gene conversion for the evolution of Y-linked duplicates have been recently investigated using analytical and simulation approaches [76, 88]. Both works considered the evolution of gene duplicates in the presence of deleterious mutations and examined how gene conversion affects the probability of fixation of new duplicates and preservation of duplicated genes once they are fixed. Both studies find that gene conversion does not enhance the probability of duplicate fixation, and, unless there are direct fitness benefits of having a duplicate (e.g., increase in dose), the fixation of Y-linked duplicates is expected to occur by drift [76, 88]. However, once duplicates are fixed, gene conversion can effectively counteract the degeneration of the Y chromosome. Gene conversion exerts its effect through regeneration of the least-mutated haplotype allowing for more efficient removal of deleterious mutations and reducing the chance that the least-mutated class will be lost by drift. These benefits of gene conversion are higher when the rate of gene conversion and the total mutation rate are high and the fitness effects of deleterious mutations are small [88]. The advantage of gene conversion can be further extended to cases where the deleterious effect of a mutation in one copy is masked by another functional copy. In this situation, selection is inefficient in removing these mutations (effectively recessive deleterious mutations). Gene conversion can expose such mutations to selection, preventing accumulation of deleterious mutations that would otherwise eventually lead to the loss of the functional copy [88]. High rates of gene conversion observed on the human Y palindromes that maintain nearly identical copies [77] might have been favored to allow efficient selection against recessive deleterious mutations. 

The results of the above studies highlight the beneficial effect of gene conversion on the removal of deleterious mutations (i.e., protection against further degeneration). But gene conversion between members of a gene family can also have the complementary effect of increasing the fixation rate of beneficial mutations. The effect of gene conversion on the rate of adaptive evolution in gene families has been investigated by Mano and Innan [91]. Using analytical and simulation approaches the authors studied the dynamics of a beneficial mutation that initially occurs in one member of the gene family and eventually spreads to all members through gene conversion reaching fixation. They show that gene conversion increases the effective population size by a factor that is equal to the size of the gene family. This leads to a higher fixation rate of beneficial mutations and a lower fixation rate of deleterious mutations in multigene families [91]. This result holds in cases with or without crossing over and should be applicable to gene families on the Y chromosome [92] although the effects are expected to be smaller due to reduced population size and the haploid nature of the Y. 

Mano and Innan's model [91] might provide a better fit to Y chromosome data than models that consider the effect of gene conversion in the presence of deleterious mutations only. The common feature of the Y chromosome across different species is the peculiar composition of its gene content with respect to function and expression. With few exceptions, genes can be divided into two broad categories: there are single-copy genes that are expressed broadly and multicopy genes that are expressed predominantly in testis and have functions related to male fertility. Furthermore, testis-expressed gene copies within gene families share high sequence identity as a result of intrachromosomal gene conversion that occurs within ampliconic regions. Given all the data that has accumulated over the years demonstrating adaptive evolution of genes with reproduction-related functions [93–98], it would not be surprising if the same pattern was found for Y-linked spermatogenesis genes. What differentiates a nontestis gene from a testis gene is the fraction of sites that can receive beneficial mutations. When in a single-copy state, the adaptive evolution of a testis gene is compromised by linkage to deleterious mutations [99]. When duplicated, gene conversion allows for more efficient removal of deleterious mutations and the beneficial mutation can now occur on a chromosome with fewer deleterious mutations [76, 88, 91]. While the fixation rate of beneficial mutations that occur anywhere on the Y is expected to increase in the presence of gene conversion, the adaptive evolution of duplicated testis genes is further accelerated by gene conversion that facilitates the spread of beneficial mutations between paralogs as described by Mano and Innan [91]. In this scenario, ampliconic/palindromic structure is maintained because it allows rapid adaptive evolution of testis genes.

In the absence of beneficial mutations fixation of duplicates occurs by drift unless the duplicate has an immediate fitness benefit associated with the increased dosage of gene product. The effect of gene conversion on the fixation of Y-linked duplicates in the presence of both beneficial and deleterious mutations has not been modeled, but it is interesting to note that gene conversion can slow down the loss of redundant duplicates [88], thereby increasing the time period during which functional duplicates are segregating in a population. This effect of gene conversion is expected to increase the chance of duplicate fixation where the direct fitness benefit is supplied by the beneficial mutations that improve gene function. The differences in the target size for beneficial mutations between nontestis and testis genes may help explain the fixation of duplications containing testis genes. Let us consider first the case of a testis gene. Duplication of a testis gene would immediately double the rate of incoming beneficial mutations. If a beneficial mutation occurs while a duplicate is segregating, gene conversion is expected to enhance the fixation of the duplicate by spreading the beneficial mutation among paralogs and by freeing beneficial mutation from its association with deleterious mutations within the ampliconic region thereby increasing the fitness of the Y chromosome that carries the duplicated genes. Duplication here can be viewed as a modifier of recombination that is under direct positive selection when a beneficial mutation occurs in one of the copies. While a duplication event will also immediately double the rate of deleterious mutations, efficient selection on a haploid chromosome and a high rate of gene conversion are expected to efficiently remove them [39, 76, 88, 91]. In the case of X-degenerate nontestis genes, mutations are less likely to have a beneficial effect as they are broadly expressed and gene conversion would only bring the potential benefit of a reduced rate of fixation of deleterious mutations. This beneficial effect might not be enough to offset the deleterious effects of ectopic crossing over between gene duplications [78, 88].

It has been also proposed that ampliconic regions have evolved gradually as the fixation of large duplications is extremely unlikely when the benefits of gene conversion associated only with the removal of deleterious mutations are considered [76]. However, the analyses of ampliconic sequence in primates suggest that some of the steps in the evolution of palindromes may involve duplication of large regions [89, 100]. Furthermore, new genes are not always acquired gene by gene; in bovine MSY, a new testis gene family has been acquired by “gene block” transposition from an autosome [46]. The proposed-above dependence of the duplicate fixation on the presence of gene conversion and adaptive mutations suggested for the testis-specific genes might also allow for fixation of large-scale duplications.

**Figure 5:**
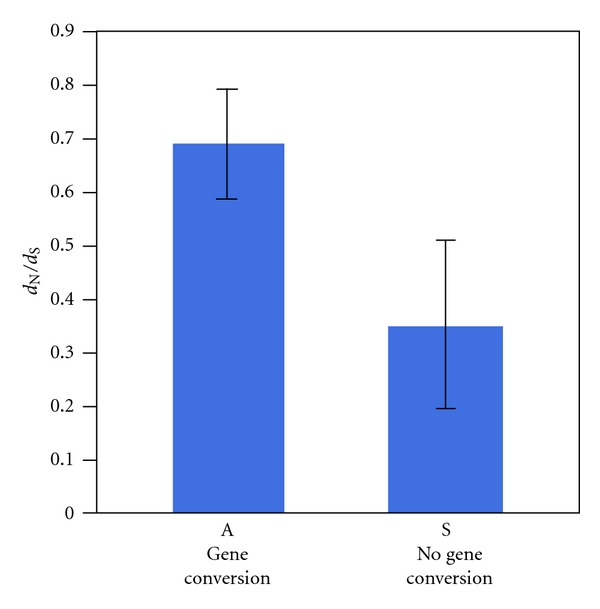
*d*_N_/*d*_S_ comparison between ampliconic (A) and single-copy (S) genes in the human-rhesus Y chromosome ([26]; Mann-Whitney test, *Z* = 3.75, *P* = 0.0002). If a gene is ampliconic in one species and not in another, it was counted as ampliconic in this comparison. Error bars indicate 95% confidence interval.

**Figure 6:**
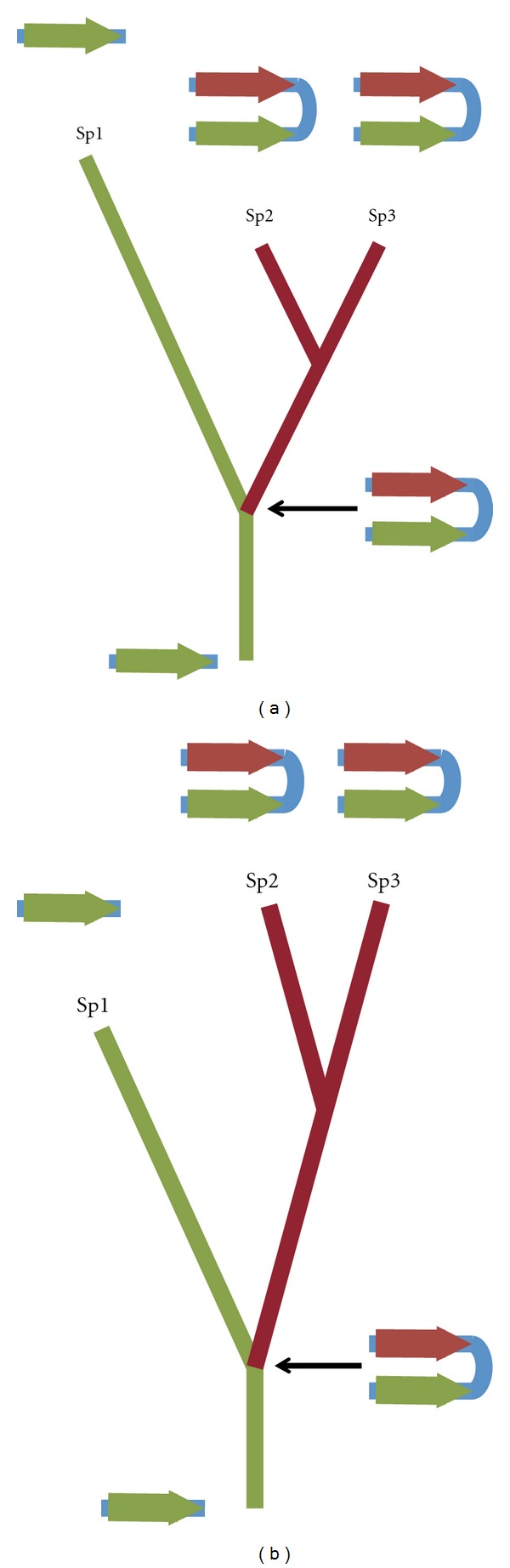
Two models of the evolution of gene families on the Y chromosome under concerted evolution. The length of the branches shown is proportional to *d*_N_/*d*_S_ ratio. (a) When gene conversion does not increase the fixation rate of beneficial mutations in multigene families, the rate of evolution is reduced compared to that of single-copy gene because gene conversion is expected to reduce the fixation rate of deleterious mutations. (b) When gene conversion increases the fixation rate of beneficial mutations in multigene families, the rate of evolution is higher compared to single-copy genes.

A prediction of the model of Mano and Innan [91] is that the rate of evolution of multicopy genes located in regions undergoing gene conversion (palindromes) should be higher than the rate of evolution of single-copy genes if they are evolving under positive selection [91, 92]. Alternatively, if adaptive evolution in testis genes is rare, the main consequence of gene conversion (and consequently, palindrome presence) would be increased efficiency of purifying selection, leading to reduced rate of evolution in multicopy genes compared to single-copy genes. This comparison is analogous to that between genes in regions of high and low recombination [97]. Comparing human and rhesus macaque Y-linked genes (data from [26]), genes in ampliconic regions show accelerated rate of evolution, with higher ratio of nonsynonymous to synonymous substitution rates compared to single-copy X-degenerate genes (Figure 5). This result might be interpreted as indicative of adaptive evolution in testis genes. However, given differences in expression profiles between the two classes of genes and the fact that rates of protein evolution correlate negatively with expression levels and not only with expression breadth [101, 102], further analyses are needed to remove the effect of gene expression on the rates of evolution. A more adequate way to test the model of Mano and Innan [91] is to look for an acceleration or deceleration of the rate of evolution in genes with the same function and expression by comparing lineages where gene is present in many copies to the lineages where the gene still remains a single-copy gene (Figure 6). Among Y-linked genes in primates (Table 1), there is one gene (RBMY) that at first glance satisfies these conditions. RBMY is a single-copy gene in rhesus macaque but has 6 copies in humans and chimps. However, the single-copy status of RBMY is a derived state as RBMY is present in multiple copies in nonprimate species [103]. We should therefore wait until data from more species becomes available to directly test the effects of gene conversion on the adaptive evolution of Y-linked gene families.

More generally, it would be expected that fast-evolving genes should be members of gene families that undergo high levels of gene conversion because ampliconic structures accelerate the rate of adaptive evolution by permitting high levels of gene conversion. A whole genome analysis of palindromes in the human genome revealed that palindromes are not only overrepresented on the Y chromosome but also overrepresented on the X chromosome, and among those palindromes with >99% arm-to-arm identity, most contain genes with testis expression [104]. The mouse X chromosome also contains many genes showing postmeiotic expression in testis that are part of amplicons including some palindromes [105]. It has been suggested that the role of these palindromes on the sex chromosomes might be to prevent meiotic sex chromosome inactivation allowing the expression of spermatogenesis genes that reside in palindromes [104]. However, recent discovery of Z-linked amplicons with testis genes in chicken [106] argues against the role of palindromes in escaping gene silencing since it is typically the heterogametic sex that undergoes meiotic sex chromosome inactivation [107], but male chickens are ZZ. The rate of evolution or gene conversion of these testis genes has not been studied, but it is notable that amplicons are enriched for the kinds of genes that frequently evolve under positive selection [93–98]. In other instances, the genes in palindromes have functions that might be under positive selection in both sexes. These are patterns that were observed in palindromes in worms for genes that were speculated to act as antimicrobial peptides [108]. There is therefore a need for systematic studies of genes in amplicons in association with the rates of evolution in the regions undergoing gene conversion in order to evaluate the relative contribution of gene conversion to patterns of gene preservation and adaptation. 

## Figures and Tables

**Table 1:** Copy number and expression profiles of MSY genes in primates.

Origin	Gene	Rhesus	Human	Chimp	X-homolog
Stratum		Ancestral genes			

	SRY	1 (?)	1 (pT)	1 (T + S)	Yes
	RBMY	1 (T)	6 (T)	6 (pT)	Yes
1	RPS4Y1	1 (T+P)	1 (B)	1 (B)	Yes
	RPS4Y2	1 (T)	1 (B)	1 (B)	Yes
	HSFY	3 (T)	2 (T)	—	Yes

2	KDM5D	1 (B)	1 (B)	1 (B)	Yes
	TSPY	5 (T)	35 (T)	6 (pT + L + Li)	Yes

	ZFY	1 (B)	1 (B)	1 (B)	Yes
	DDX3Y	1 (B)	1 (B)	1 (B)	Yes
	UTY	1 (B)	1 (B)	1 (B)	Yes
3	EIF1AY	1 (B)	1 (B)	1 (B)	Yes
	CYorf15A	1 (B)	1 (B)	1 (B)	Yes
	CYorf15B	1 (B)	1 (B)	ps (B)	Yes
	USP9Y	1 (B)	1 (B)	ps (B)	Yes
	TMSB4Y	1 (B)	1 (B)	ps	Yes

	AMELY	1 (?)	1 (B)	1 (?)	Yes
4	NLGN4Y	1 (B)	1 (B)	1 (B)	Yes
	TBL1Y	1 (B)	1 (B)	ps (B)	Yes

5	PRKY	1 (B)	1 (B)	1 (B)	Yes
	MXRA5Y	1 (B)	ps	ps	Yes

	**Total:**	**26**	**59**	**24**	

		Added genes			

A-transposed	DAZ	2 (T)	4 (T)	4 (T)	No
A-retroposed	CDY	2 (T)	4 (T)	5 (pT)	No
	XKRY	1 (B)	2 (T)	ps (pT)	No
	BPY2	—	3 (T)	2 (T)	No
	PRY	—	2 (T)	—	No
	VCY	—	2 (T)	2 (?)	Yes
X-transposed	PCDH11Y	—	1 (Br)	—	Yes
X-transposed	TGIF2LY	—	1 (T)	—	Yes

	**Total:**	**5**	**19**	**13**	

	**Total AG** ^ a^	**12**	**60**	**25**	
	**Total AF** ^ b^	**4**	**9**	**6**	
	**Grand Total:**	**31**	**78**	**37**	

Modified from [26]. Expression data from [26, 34, 36, 49]. T: testis, pT: predominantly testis, B: broad, Br: brain, P: prostate, S: spleen, L: lung, Li: liver, ?: not known. Absent gene (—), pseudogene (ps). ^a^Ampliconic genes; ^b^Ampliconic families.
